# Quantitative analysis of the myelin *g*-ratio from electron microscopy images of the macaque corpus callosum

**DOI:** 10.1016/j.dib.2015.05.019

**Published:** 2015-06-17

**Authors:** Nikola Stikov, Jennifer S.W. Campbell, Thomas Stroh, Mariette Lavelée, Stephen Frey, Jennifer Novek, Stephen Nuara, Ming-Kai Ho, Barry J. Bedell, Robert F. Dougherty, Ilana R. Leppert, Mathieu Boudreau, Sridar Narayanan, Tanguy Duval, Julien Cohen-Adad, Paul-Alexandre Picard, Alicja Gasecka, Daniel Côté, G. Bruce Pike

**Affiliations:** a Montreal Neurological Institute, McGill University, Montreal, Canada; b Stanford University, Stanford, CA, United States; c Hotchkiss Brain Institute and Department of Radiology, University of Calgary, Calgary, Alberta, Canada; d École Polytechnique de Montréal, Montréal, Canada; e École supérieure d׳ingénieurs en génie électrique, Rouen, France; f Université Laval, Quebec City, Canada

## Abstract

We provide a detailed morphometric analysis of eight transmission electron micrographs (TEMs) obtained from the corpus callosum of one cynomolgus macaque. The raw TEM images are included in the article, along with the distributions of the axon caliber and the myelin *g*-ratio in each image. The distributions are analyzed to determine the relationship between axon caliber and *g*-ratio, and compared against the aggregate metrics (myelin volume fraction, fiber volume fraction, and the aggregate *g*-ratio), as defined in the accompanying research article entitled ‘*In vivo* histology of the myelin *g*-ratio with magnetic resonance imaging’ (Stikov et al., *NeuroImage*, 2015).

Specifications table

**Table:** 

Subject area	Neuroanatomy
More specific subject area	Morphometry
Type of data	Electron microscopy (EM) images, and figures illustrating the EM analysis
How data was acquired	FEI Tecnai 12 120 kV Transmission Electron Microscope
Data format	TIFF (raw)
Experimental factors	Specimen perfusion fixed with 2% paraformaldehyde and 2% glutaraldehyde, then stained with osmium
Experimental features	EM images were analyzed using an automated method for the segmentation and morphometry of white matter
Data source location	Montreal, Canada
Data accessibility	Data is included with this article

Value of the data

• There is a limited number of publicly available datasets for comprehensive morphometric analysis of white matter microstructure.
• Our dataset illustrates the complex relationship between axon caliber and the myelin *g*-ratio in eight distinct regions of the corpus callosum of a cynomolgus macaque.
• The data is systematized in a way that makes it easy to explore the relationship between aggregate metrics (AVF, MVF, aggregate *g*-ratio) and the underlying distributions producing these metrics.

## 1 Data, experimental design, materials and methods

### 1.1 Experimental design and analysis

One healthy cynomolgus macaque was euthanized by means of exsanguination with anesthesia provided by ketamine (15–20 mg/kg i.m.) and sodium pentobarbital (100 mg/kg i.v.). When there was an absence of reflexes, the animal was perfused transcardially with heparinized saline (0.9% NaCl and 0.5 ml/L of heparin), and then with 2% paraformaldehyde and 2% glutaraldehyde solution. 48 h later, the corpus callosum was extracted, sectioned on a vibratome at 50 µm thickness, prepared with osmium and divided into eight segments of equal length from anterior to posterior. Electron microscopy (EM) was performed on samples from each of the segments one to eight at 1900× magniﬁcation, yielding images of 21×28 µm^2^ with 9.144 nm/pixel. The images are included as supplementary data and are the same ones used in [1].

**Fig. 1:**
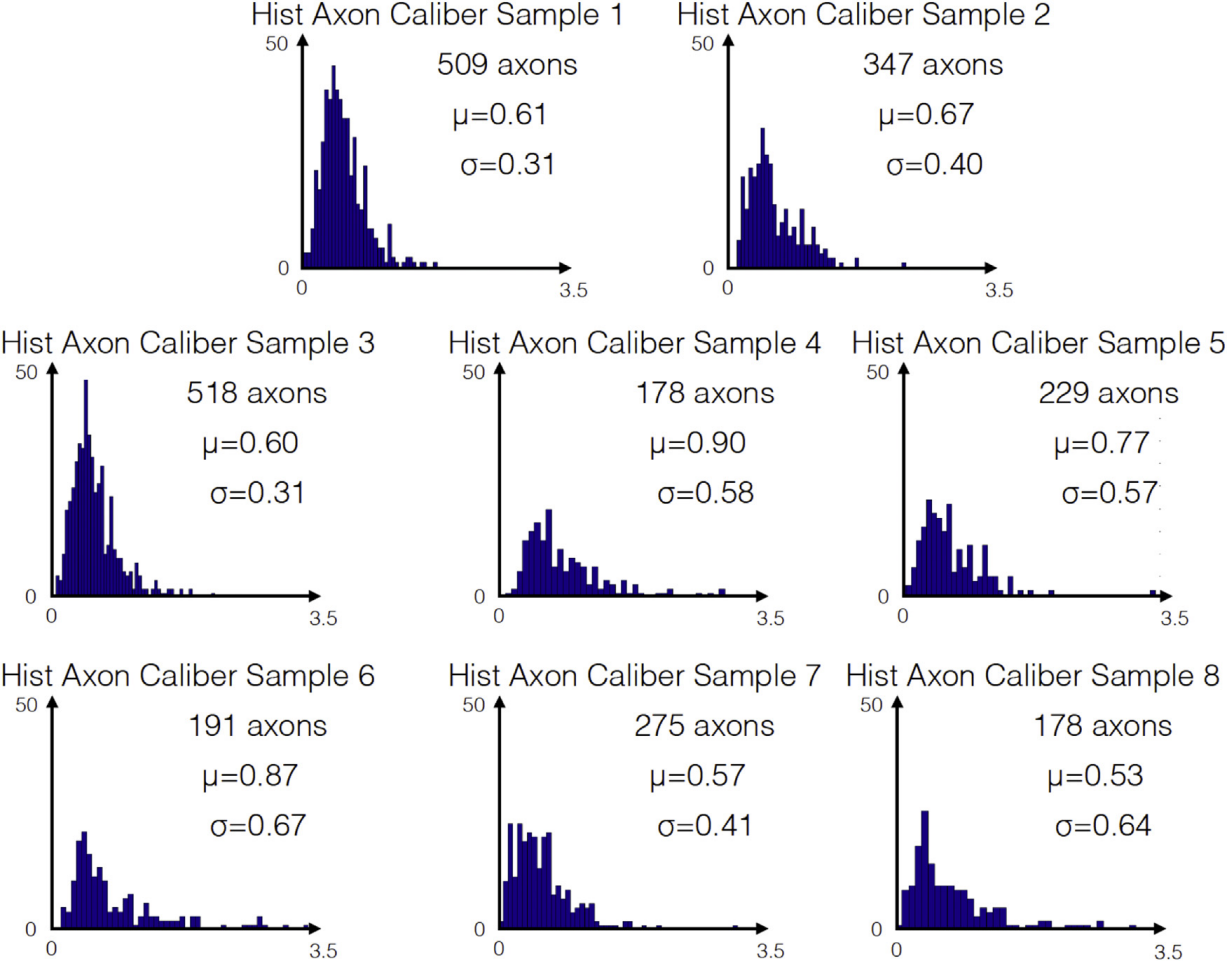
Distribution of axon caliber in images 1–8.

**Fig. 2:**
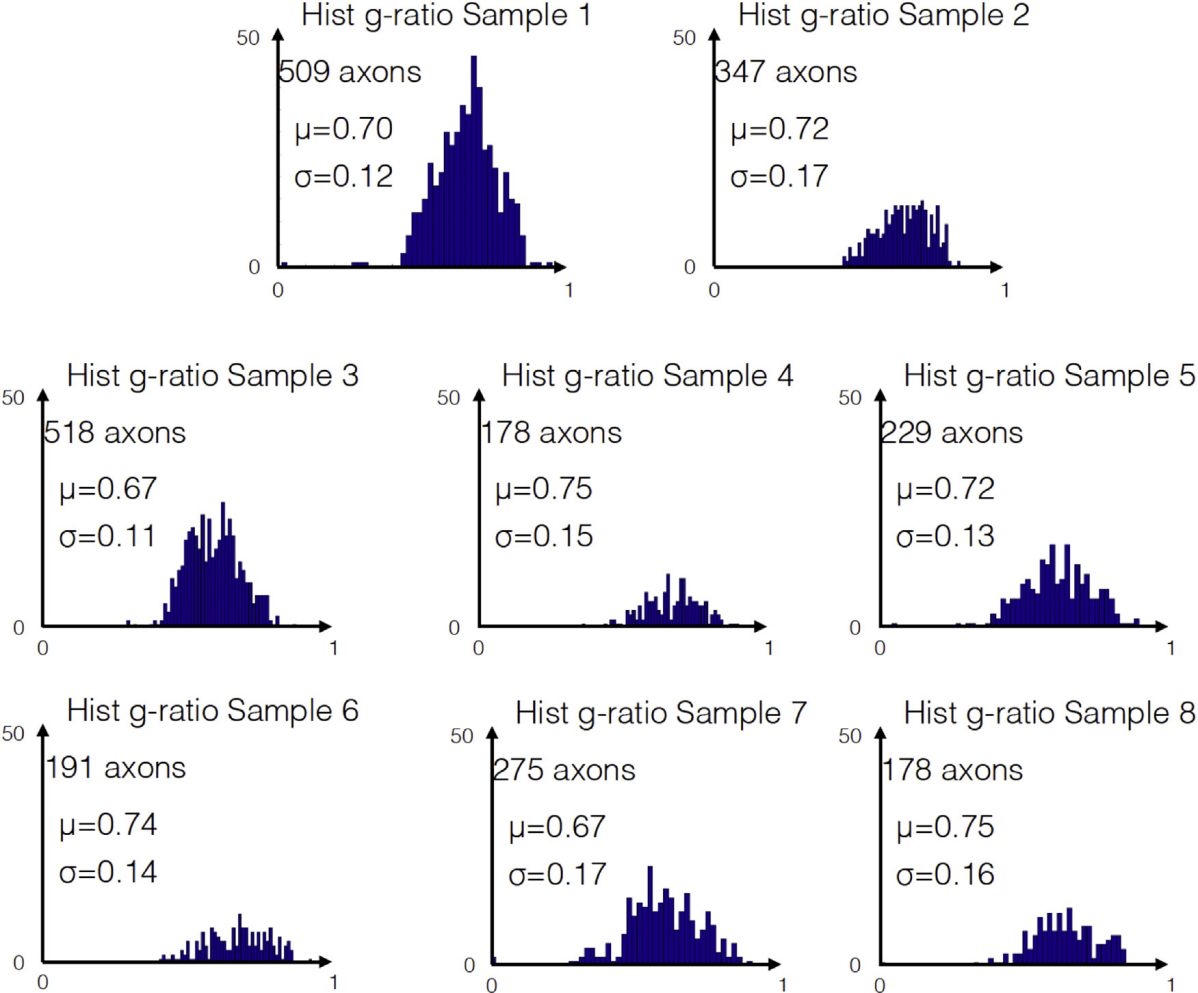
Distribution of *g*-ratios in images 1–8.

**Fig. 3:**
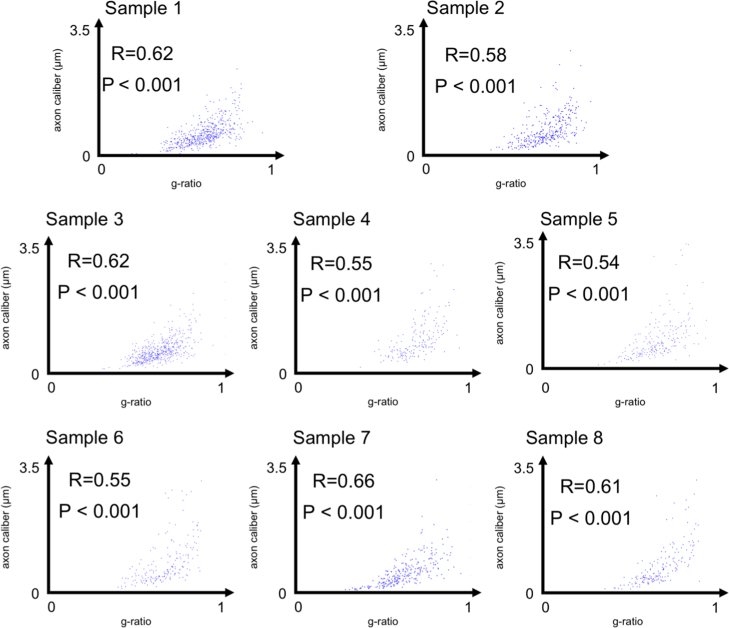
Correlation between axon caliber and myelin *g*-ratio in images 1–8.

Axon and myelin segmentation was performed on each image using an automated method for large scale histology, and details of the analysis can be found in [2]. For each EM image the software provided an axon count, the individual axon calibers and the corresponding myelin *g*-ratios. Fig. 1 shows the distribution of the axon caliber in the eight corpus callosum images, and Fig. 2 shows the distribution of the corresponding myelin *g*-ratios. While it is difficult to draw conclusions about an entire segment of the CC based on a single image, certain trends, such as large axons in the splenium of the corpus callosum, are consistent with the literature [3]. Fig. 3 shows that the myelin *g*-ratio is only moderately correlated with axon caliber, justifying the need for measuring the two quantities separately. Table 1 shows listing of aggregate metrics (MVF, FVF and aggregate *g*-ratio), as defined in [4]. For the images below, the aggregate *g*-ratio (defined as $g_{aggregate}=\sqrt{1-\;\mathrm{MVF}/\mathrm{FVF}}$) correlates with the mean *g*-ratio (*r*=0.85, *p*=0.007), even though it slightly overestimates it (*g*_*aggregate*_=1.05⁎*g*_*mean*_+0.036). We expect this relationship to remain significant in regions where the *g*-ratio is relatively uniform. These two measures will be equal if the *g*-ratio is the same for all axons, and will deviate otherwise. In extreme cases, the two measures might not correlate (see discussion in [1]).

## Figures and Tables

**Table 1:** Measurements of the MVF, FVF, and the *g*-ratio (aggregate and mean) for images 1–8. The aggregate *g*-ratio correlates significantly with the mean *g*-ratio (*r*= 0.85, *p*= 0.007). For definitions of these metrics, please consult [1].

Sample	MVF	FVF	Aggregate *g*-ratio	Mean *g*-ratio
1	0.34	0.66	0.70	0.65
2	0.27	0.56	0.72	0.66
3	0.40	0.73	0.67	0.62
4	0.22	0.50	0.75	0.68
5	0.27	0.56	0.72	0.65
6	0.26	0.56	0.74	0.66
7	0.23	0.41	0.67	0.60
8	0.19	0.43	0.75	0.65
